# *Craniomaxillofacial Trauma and Reconstruction*: A New Era in Open Access Publishing

**DOI:** 10.3390/cmtr18010001

**Published:** 2025-01-02

**Authors:** Yiu Yan Leung, Kathy Fan, Florian M. Thieringer

**Affiliations:** 1 Oral and Maxillofacial Surgery, Faculty of Dentistry, The University of Hong Kong, Hong Kong, China; mikeyyleung@hku.hk; 2 King’s College Hospital NHS Foundation Trust, London SE5 9RS, UK; kathy.fan@kcl.ac.uk; 3 King’s College London, University of London, London SE5 9RS, UK; 4 Oral- and Cranio-Maxillo-Facial Surgery, University Hospital Basel, CH-4031 Basel, Switzerland; 5 Swiss MAM Research Group, Department of Biomedical Engineering, University of Basel, CH-4123 Allschwil, Switzerland

*Craniomaxillofacial Trauma and Reconstruction* (CMTR). Our journal has found a new home with the open access publisher MDPI. As the journal’s Editors-in-Chief, we are very excited about the possibilities afforded by this new relationship. CMTR is and will remain 100% owned by AO CMF, a global multispecialty community of clinicians dedicated to promoting excellence in OMFS patient care, facial trauma, and musculoskeletal disorders outcomes. Our partnership with MDPI considerably expands our horizons.

A pioneer in scholarly, open access publishing, MDPI has supported academic communities since 1996. Publishing more than 450 fully open access titles (https://www.mdpi.com/about/journals, accessed on 12 November 2024), MDPI supports more than 800 academic institutions worldwide, helping them adhere to national mandates while facilitating the publication of authors’ papers in fully compliant (CC BY) open access journals. As of December 2023, 26 Nobel laureates contributed to over 75 articles across 25 MDPI journals. We are delighted to be with such an excellent company. We are sure that it will inspire us to more remarkable achievements in our fast-developing field of craniomaxillofacial surgery.

Our commitment to our objectives is unchanging, although we have a new home. Our confidence that we can achieve them has, however, grown. Drawing on the resources of a world expert in open access publishing, we now have the opportunity to make the best and most promising craniomaxillofacial research available to scholars globally without restriction. With rigorous ethical and editorial standards and a robust and efficient editorial process, our new publishing partner offers us a platform for growth that has the potential to take craniomaxillofacial surgery as a discipline to new levels of effectiveness and reliability, permitting surgeons to deliver invaluable benefits to patients worldwide whose lives are impacted by conditions that can only be treated by expert surgical interventions. This is an exciting moment for our entire scientific and medical community.

As CMTR’s three Editors-in-Chief, we will work closely with our outstanding 50 Associate Editors and our Editorial Board (https://www.mdpi.com/journal/cmtr/editors, accessed on 12 November 2024). We hope and trust that our Editorial Board will grow with the evolution of CMTR itself, which will additionally publish Special Issues on selected topics of critical relevance. 

We hope you will enjoy this first issue of CMTR in its new format, and we welcome your papers for consideration and suggestions for how the journal can be further developed to better support our readership and the patients we all serve.

We look forward to hearing from you!

*Professor Mike Yiu Yan Leung*

*Professor Kathy Fan*

*Professor Florian M. Thieringer*

Editors-in-Chief

